# Sex-Related Difference in Nitric Oxide Metabolites Levels after Nephroprotectant Supplementation Administration against Cisplatin-Induced Nephrotoxicity in Wistar Rat Model: The Role of Vitamin E, Erythropoietin, or N-Acetylcysteine

**DOI:** 10.5402/2013/612675

**Published:** 2013-03-11

**Authors:** Mehdi Nematbakhsh, Zahra Pezeshki

**Affiliations:** ^1^Water and Electrolytes Research Center, Isfahan University of Medical Sciences, Isfahan 81745, Iran; ^2^Department of Physiology, Isfahan University of Medical Sciences, Isfahan 81745, Iran

## Abstract

*Background*. Nitric oxide (NO) concentration in serum is altered by cisplatin (CP), and NO influences CP-induced nephrotoxicity. The effect of nephroprotectant agent supplementation (vitamin E, human recombinant erythropoietin (EPO), or n-acetylcysteine (NAC)) on the NO metabolites levels after CP administration in the two genders was determined. *Methods*. Sixty-four adult Wistar rats were randomly divided into 10 groups. Male and female rats in different groups received vehicle (saline), CP (7 mg/kg) alone, CP plus EPO (100 IU/kg), CP plus vitamin E (250 mg/kg), and CP plus NAC (600 mg/kg). CP was administrated as a single dose, but the supplementations were given for a period of 7 days. *Results*. In male rats, the serum levels of total NO metabolites (NO_*x*_) and nitrite were increased significantly (*P* < 0.05) by CP. However, vitamin E significantly reduced the serum levels of these metabolites, which was increased by administration of CP (*P* < 0.05), and such findings were not observed for female rats. The EPO or NAC did not influence NO metabolites neither in male rats nor in female rats. *Conclusion*. Although vitamin E, EPO, and NAC are reported to be nephroprotectant agents against CP-induced nephrotoxicity, only vitamin E could reduce the level of all NO metabolites only in male rats.

## 1. Introduction

Cisplatin (CP) is the most common antitumor drug in clinic. The most common side effect of CP is nephrotoxicity. However, hepatotoxicity and testicular toxicity are also frequently observed. CP is a platinum compound, which is accompanied by decrease in glomerular filtration rate (GFR), increase in blood urea nitrogen (BUN) and serum levels of creatinine, and tubular injury [1–3]. CP may disturb endothelium and endothelial function [4–7]. Nitric oxide (NO) is a marker of endothelial function. However, some studies documented that administration of CP increases the serum level of NO [8, 9], and on the other hand, increase of NO level may promote CP-induced nephrotoxicity [8]. NO is synthesized from the amino acid L-arginine by the endothelial NO synthase (eNOS) in endothelium. It is documented that L-arginine as precursor of NO has protective role against CP-induced nephrotoxicity [1, 3]. Some paradoxes could be seen here; CP may increase NO [1, 2], NO may promote CP-induced nephrotoxicity [8], and NO donates agents attenuating CP-induced nephrotoxicity [10].

NO is not a stable molecule, and its half life in circulation is considerably short, but it is rapidly oxidized to nitrite and nitrate that are considered as NO metabolites. The sum of NO metabolites called NO_*x*_, and all NO_*x*_, nitrite (NO2^−^), and nitrate (NO3^−^) are considered as endogenous NO products [11, 12]. However, it has been shown that nitrite is a good marker for endothelial NO production, while plasma nitrate levels are influenced by a variety of NOS-independent factors [12–15]. Among the NO metabolites, nitrite is a major oxidative metabolite, which was implicated to be an indicator of NOS activity. It has been shown that up to 70%–90% of plasma nitrite is derived from eNOS activity in fasted subjects [16]. Furthermore, NOS inhibition in humans, pigs, dogs, and mice significantly decreases the plasma nitrite concentration [17]. Another point is gender. NO production is also reported to be gender related [18–23], and accordingly, it is important to know which types of NO metabolites form NO system (nitrite, nitrate, or NO_*x*_) during CP therapy and which types of NO metabolites are influenced by CP in the two sexes.

In order to attenuate CP-induced nephrotoxicity, many nephroprotectant agents such as vitamin E, human recombinant erythropoietin (EPO), and n-acetylcysteine (NAC) were subject of research in different models [24–33]. Therefore, in the present study, we attempt first to find which metabolite, nitrite, nitrate, or NO_*x*_, is disturbed by CP in the two genders and second to determine whether the protective role of vitamin E, EPO, or NAC against CP-induced nephrotoxicity is accompanied by changes of nitrite or nitrate levels.

## 2. Methods and Materials

### 2.1. Animals

The investigation was performed on 64 adult male (175–200 g) and female (150–180 g) Wistar rats (Animal Centre, Isfahan University of Medical Sciences, Isfahan, Iran). The rats were housed at a temperature of 23–25°C and had free access to water and rat chow. The research protocols were in advance approved by the Isfahan University Medical Sciences Ethics Committee.

### 2.2. Drugs

CP (cis-Diammineplatinum(II) dichloride, code P4394) and vitamin E from Sigma (St. Louis MO, USA), EPO from Janssen-Cilag (Czech Republic), NAC, as Flumil Antidote 20% from Pharmazam S. A. (Barcelona, Spain) were purchased.

Flumil Antidote 20% is the commercial name of NAC.

### 2.3. Experimental Protocol

The animals were randomly divided into 10 experimental groups. Groups 1 (*n* = 7) and 2 (*n* = 7) were assigned as male and female negative control groups that received vehicle (saline) alone during the study. Groups 3 (*n* = 7) and 4 (*n* = 7) were assigned as male and female positive control groups that received single dose of CP (7 mg/kg) and then were treated with vehicle (~0.5 mL/day) every day for one week. Groups 5 (male, *n* = 6) and 6 (female, *n* = 6) received single dose of CP and then were treated with EPO (100 IU/kg/day, i.p.) every day for one week. The other groups received the same regimen as groups 5 and 6, except for vitamin E (250 mg/kg) instead of EPO, groups 7 (male, *n* = 6) and 8 (female, *n* = 6) or NAC (600 mg/kg), groups 9 (male, *n* = 6) and 10 (female, *n* = 6).

Seven days after CP administration, blood sample was obtained and the serum was collected from each blood sample and stored at −20°C until measurements. 

### 2.4. Measurements

The NO stable metabolites (nitrite/nitrate, NO_x_) were measured in serum by an ELISA assay kit (Cayman Chemical Co.) that involves the Griess reaction.

### 2.5. Statistical Analysis

Data are expressed as mean ± SEM. The groups were compared by one-way analysis of variance (ANOVA) with regard to the serum levels of NO_*x*_, nitrite, and nitrate. Post hoc testing was performed for intergroup comparisons using the least significant difference (LSD) test. *P* values <0.05 were considered statistically significant.

## 3. Results

### 3.1. Effect of CP on Serum NO_*x*_, Nitrate, and Nitrite Levels

The serum levels of NO_*x*_ and nitrite were increased significantly (*P* < 0.05) by CP administration in male, while such finding was not obtained in female (Table 1). The data also indicated that the serum level of nitrate was increased by CP administration nonsignificantly. This finding revealed that alteration of serum NO metabolites (NO_*x*_ and nitrite) after CP injection is gender related.

### 3.2. Effect of EPO, Vitamin E, and NAC on Serum NO_*x*_, Nitrate, and Nitrite Levels Increased by CP

**Figure 1:**
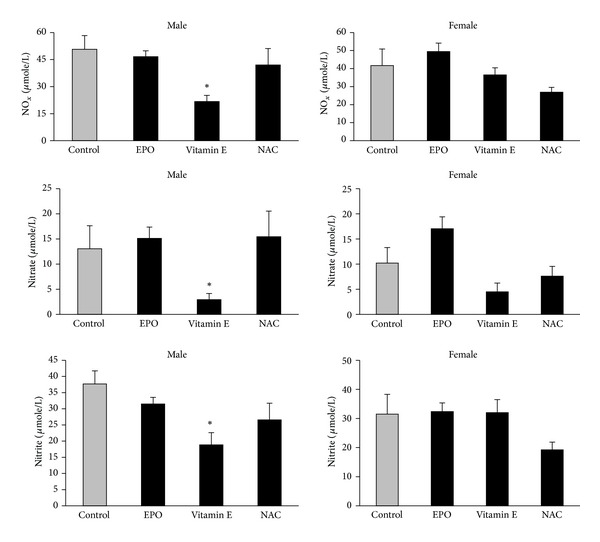
Effect of EPO, vitamin E, and NAC on CP-increased serum levels of NO_*x*_, nitrate, and nitrite in male and female rats. The EPO, vitamin E, and NAC groups were compared with positive control group. (*) shows significant difference from control group (*P* < 0.05).

The data are presented in Figure 1. In male, vitamin E significantly reduced the serum levels of NO_*x*_ and nitrite, which were increased by CP administration (*P* < 0.05, Figure 1), while this was not observed in female. Although EPO and NAC are considered as nephroprotectant agents against CP-induced nephrotoxicity, these agents potentially could not reduce the CP-increased serum levels of NO_*x*_ and nitrite neither in male nor in female. This finding revealed that vitamin E is a more potent nephroprotectant agent in reducing nephrotoxicity via reduction of NO metabolites. However, this potential effect of vitamin E is gender dependent.

## 4. Discussion

The main findings of this research were as follows: NO_*x*_ and nitrite are increased by CP administration and vitamin E as a nephroprotectant agent reduces the NO metabolites. Since NO may promote CP-induced nephrotoxicity [8], it is important to know which one of the NO metabolites is suitable to monitor the NO bioavailability [12, 34], and also it is important to select the appropriate nephroprotectant supplementation to modulate NO metabolite levels after CP administration. It is well known that the levels of nitrate and total NO_*x*_ do not vary during pharmacological modulation of L-arginine-NO pathway [15], and oxidation of endogenous NO is one of the most important endogenous sources of NO_*x*_ [16]. Endogenous NO production is performed by NOS isoforms: constitutively active forms of eNOS, neuronal NO synthase (nNOS), and the inducible isoforms (iNOS). The relevant amount of NO in circulation is not from nNOS [35]. The iNOS is expressed by smooth muscle cells and macrophages [36] and may contribute to NO production under pathological conditions. However, selective inhibition of iNOS reduces CP-induced histological damage, renal dysfunction, oxidative stress, and nitrosative stress [37]. It is reported that nitrite is a suitable marker of NO formation [12], and variation of NOS activity is associated with direct parallel alteration of nitrite concentrations [13, 14] while up to 70%–90% of plasma nitrite is derived from eNOS activity [16]. Furthermore, NOS inhibition in humans, pigs, dogs, and mice significantly decreases the plasma nitrite concentration [17]. Therefore, although nitrite is the most important NO metabolite to monitor NO formation, it seems that vitamin E provides its antioxidant effect against CP-induced nephrotoxicity by reduction of both nitrite and nitrate in male, but not in female. Gender-related nitrate and nitrite levels have been reported in rat brain [38]. Watanabe et al. measured the serum levels of NO metabolites in 263 healthy subjects and obtained some different results for male and female subjects. Furthermore, the NO_*x*_ concentration was affected by age in women [39]. Other studies also demonstrated gender-related characteristics of NO system [18–20, 40–45]. Some other studies also reported the sex-based difference of CP-induced nephrotoxicity [46–49]. Therefore, the influence of gender on both CP-induced nephrotoxicity and NO metabolite could be responsible for sex-based difference of NO metabolite change when supplementations such as vitamin E, EPO, or NAC are administrated to protect the kidney during CP therapy.

## 5. Conclusion

Although all vitamin E, EPO, and NAC have potentially antioxidant effect to be nephroprotectant against CP-induced nephrotoxicity, vitamin E could reduce the level of all NO metabolites which possibly are promotion marker of kidney toxicity after CP administration.

## Figures and Tables

**Table 1:** Effect of CP on serum NO_x_, nitrate, and nitrite levels in male and female rats. The data was compared between negative and positive control groups using Student's *t*-test.

Factors		NO_x_ (*μ*mole/L)	*P*	Nitrate (*µ*mole/L)	*P*	Nitrite (*µ*mole/L)	*P*
Gender	Group						
Male	1 (negative control)	22.43 ± 3.0	0.005	9.23 ± 1.67	0.45	13.21 ± 2.18	0.00
	3 (positive control)	50.71 ± 7.54		13.03 ± 4.58		37.68 ± 4.04	

Female	2 (negative control)	26.22 ± 2.02	0.13	6.50 ± 1.83	0.32	19.71 ± 1.03	0.12
	4 (positive control)	41.68 ± 9.17		10.20 ± 3.07		31.48 ± 6.83	
